# Absence of the Labiomental Groove: A Common but Preventable Unpleasant Aesthetic Problem of the Lower Lip-Chin Burn Reconstruction

**Published:** 2017-09

**Authors:** Ali Akbar Mohammadi, Soheil Mohammadi

**Affiliations:** 1 Burn and Wound Healing Research Center, Plastic and Reconstructive Surgery Ward, Shiraz University of Medical Sciences, Shiraz, Iran;; 2 Tehran University of Medical Sciences, Tehran, Iran

**Keywords:** Labiomental groove, Lower facial burn, Lower lip burn, Chin burn

**DEAR EDITOR**

The labiomental groove is the deepest point of contour change at the junction of the lower lip and chin. It defines the sublabiale point. The labiomental groove is located at about one third the distance from stomion to gnathion. It represents the level of vestibule, but, more pertinently, it is the superior attachments of the mentalis muscle. The chin composes about two thirds of this part of lower face, thus forming a ratio of 1:2, lower lip to chin.^1^ The depth of the labiomental groove is 4-6 mm and it is deeper for men than women.^2^

Also labiomental angle is roughly about 120 degrees. In non-burned patient, decreased lower face, can deepen the depth of this groove. But in lower face burn patients, there is decreased depth or absence of labiomental groove in spite of decreased facial height due to scar contracture, flattened mental prominence and thick lower lip.^2^^,^^3^ In lower lip- chin reconstruction, excision of lip–chin aesthetic unit and adequate release of the lower lip to completely cover the lower teeth, is performed and in the case of thick lower lip and a flattened mental prominence, debulking of lower lip, sculpting of labiomental groove and even chin implant insertion are recommended. The lip-chin unit is then resurfaced with full thickness or thick split thickness skin graft.^2^^,^^4^ But, what we see in a significant number of reconstructed lower faces is somewhat acceptable appearance of lip–chin unit in frontal view, but aesthetically unacceptable contour in lateral view (Figure 1), which is due to decreased depth or absence of labiomental groove. This happens due to some skin graft contraction, fibrosis and persistent tissue edema, in spite of some sculpturing of labiomental groove.^2^^,^^5^^,^^6^ We think it is mostly due to inadequate labiomental groove sculpturing by burn surgeons. 

**Fig. 1A-D:**
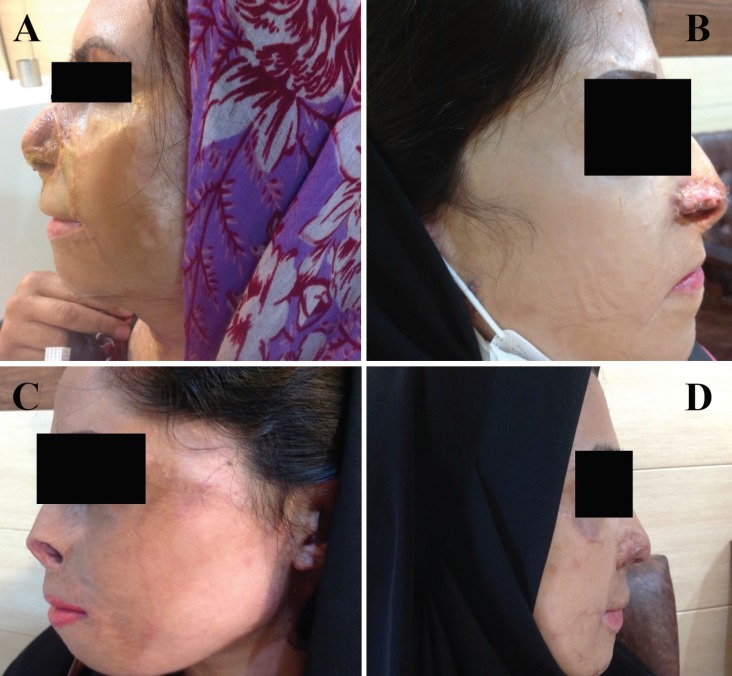
Aesthetically unacceptable appearance in lateral view, due to decreased depth or absence of labiomental groove

In our experience, to prevent this unpleasant aesthetic complication, in addition to adequate release of lip and chin fat pad, significant overcorrection at the junction of lower lip-chin unit with excision of almost all burned and nonburned tissues over the lower part of orbicularis oris and upper part of mentalis muscle (except a very thin tissue) was needed (Figure 2), and there is better aesthetic results with this modification (Figure 3). 

**Fig. 2:**
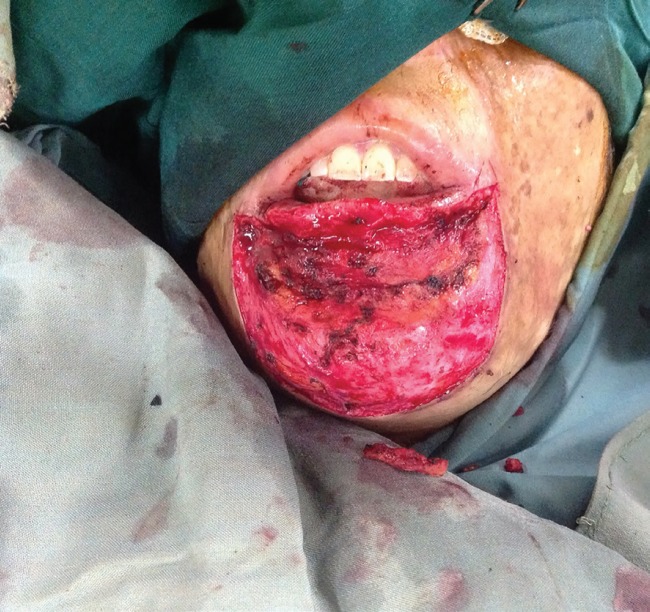
Excision of almost all burned and non-burned tissues over the lower part of orbicularis oris and upper part of mentalis muscle

**Fig. 3:**
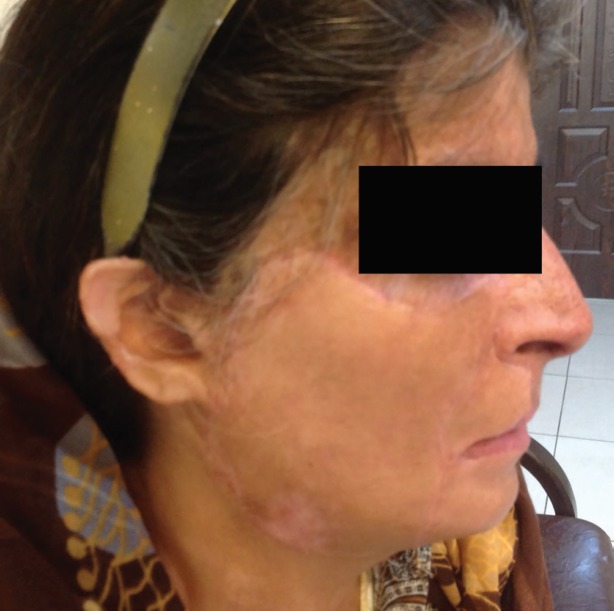
Aesthetically acceptable appearance in lateral view, due to adequate depth of labiomental groove.

## CONFLICT OF INTEREST

The authors declare no conflict of interest.

## References

[1] Farkas LG, Sohm P, Kolar JC, Katic MJ, Munro IR (1985). Inclinations of the facial profile: art versus reality. Plast Reconstr Surg.

[2] Rosen HM (1991). Aesthetic refinements in genioplasty: the role of the labiomental fold. Plast Reconstr Surg.

[3] Singh GD, Clark WJ (2003). Soft tissue changes in patients with Class II Division 1 malocclusions treated using Twin Block appliances: finite-element scaling analysis. Eur J Orthod.

[4] Singh GD (2002). Morphospatial analysis of soft-tissue profile in patients with Class II Division 1 malocclusion treated using twin block appliances: geometric morphometrics. Orthod Craniofac Res.

[5] Wei FC, Tan BK, Chen IH, Hau SP, Liau CT (2001). Mimicking lip features in free-flap reconstruction of lip defects. Br J Plast Surg.

[6] Manafi A, Pooli AH, Habibollahi P, Saidian L (2009). Retrusive chin reconstruction after burn injuries using submental and labiomental fat flaps: an innovative method. Eplasty.

